# An Examination of Multicultural Parents’ Understanding of Supporting Their Children’s Creativity

**DOI:** 10.3390/bs16050826

**Published:** 2026-05-20

**Authors:** Esra Kantar Muslu, Eda Yazgin

**Affiliations:** 1 Faculty of Educational Sciences, Final International University, 99320 Kyrenia, Cyprus; esra.muslu@final.edu.tr; 2 Faculty of Education, Eastern Mediterranean University, 99628 Famagusta, Cyprus

**Keywords:** creativity and cultural influences, creativity and multiculturalism, creativity in children, multicultural parents

## Abstract

This study explores how multicultural parents support their children’s creativity and examines the influence of cultural factors on this process. The study employed a qualitative cultural analysis/ethnography approach to examine in depth the multicultural parents’ perspectives on supporting their children’s creativity and how they evaluate and encourage creativity within their cultural context. The study group consisted of multicultural parents with children aged 36–72 months living in the Turkish Republic of Northern Cyprus. Data were collected using the three tools. The first was a researcher-developed form to provide participants’ demographic information. The second was a researcher-developed semi-structured Interview Form. Finally, the researchers conducted home visits, and collected relevant visual and written materials related to the activities, shared by parents to enhance data diversity. Content analysis was used to examine the collected visual and written records. The findings indicated that multicultural parents supported their children’s creativity through various cultural activities both at home and in external social environments. They encouraged their children to assume various roles to promote active engagement in cultural activities. Children were not only spectators but were also active participants. Furthermore, the interaction of diverse cultures contributed to the formation of new ideas and perspectives in children’s minds.

## 1. Introduction

Individuals begin engaging in intense interaction with their cultural environment from birth. Parents, as the first and most fundamental source of information, pass on the cultural family heritage to their children throughout this process. This transmission occurs across multiple dimensions—such as language, values, norms, traditions, customs, and beliefs—and also supports children’s creativity (Kim & Park, 2020). Children can develop new ideas and approaches by combining their original thoughts and imagination with the cultural elements they learn. In this case, cultural learning goes beyond the knowledge transfer and provides a significant contribution that includes creativity in the individual’s identity formation processes (Saad et al., 2013). The transmission of family culture forms the foundation of individuals’ lifelong cultural adaptation. However, creativity allows individuals to reshape and redevelop their cultural heritage during this adaptation process (Rogoff, 2003). The context of a multicultural family further enriches cultural transmission. Parents from different cultural backgrounds combine their values, traditions, and customs and present them to their children. This enables children to gain a multifaceted and rich cultural experience (Seider et al., 2023). Children raised in multicultural families develop intellectual flexibility by being exposed to different perspectives and lifestyles (Martinez, 2012). When multicultural parents pass on their cultural heritage to their children, they not only transmit knowledge, values, celebrations, and rituals, but also encourage children to use this heritage creatively. Children develop new ideas and approaches by combining the different cultural elements they learn with their original thoughts and imagination. This cultural diversity and richness support and develop children’s creative thinking skills by encouraging them to consider multiple perspectives. Leung et al. (2008) reported that multicultural experiences encouraged the creative expansion of ideas. They highlighted that multicultural experiences could contribute to creative expansion in at least five ways. First, multicultural experiences expose individuals to a variety of scenarios, ideas, concepts, and situations, increasing the possibility of generating new combinations in creative processes. Second, understanding that the same behavior can have different functions and meanings across cultures develops individuals’ ability to recognize different cultural dynamics. Third, adopting and adapting alternative concepts to new environments leads individuals’ cognitive structures to become unstable and allows them to access unfamiliar information. Fourth, individuals who are skilled at gathering ideas from different sources and applying them creatively can leverage the advantages of having multicultural experiences. Finally, the process of resolving incompatible ideas leads to greater cognitive complexity in individuals with multicultural experiences, which in turn creates a higher likelihood of creativity.

Previous studies have mostly focused on the perceptions of creativity among families from different cultures and the impact of these perceptions on children’s creativity levels. However, this study examines how multicultural families pass on cultural values while raising their children and how these transmitted cultural values support their children’s creativity. This study aims to contribute to the literature by offering a wide perspective on children and creativity in the context of multiculturalism, cultural differences, and values.

Another aim of this study is to explore how multicultural parents support their children’s creativity and examine the influence of cultural factors on this process. Within this basic scope, the study seeks to answer the following research questions:

1. What does creativity mean for parents?
2. What do parents do to support their children’s creativity?
3. What values and messages do parents aim to convey through the activities they engage in with their children, which represent their culture?
4. How do parents’ attitudes, such as incorporating traditional elements representing their culture in activities they perform with their children, reflect on their child-rearing approaches?

## 2. Materials and Methods

### 2.1. Research Model

The study employed a qualitative research method to closely examine multicultural parents’ reflections on their understanding of how to support their children’s creativity. This method is appropriate for understanding and interpreting participants’ experiences, opinions, understandings, beliefs, and feelings (Büyüköztürk et al., 2022). The cultural analysis/ethnography-design study aimed to reveal how participants evaluate, support, or limit creativity within a cultural context. This design sought to provide an in-depth look at participants’ daily lives by examining their behaviors, rituals, language use, and cultural symbols. The ethnographic design was determined to be the most appropriate for this study, as culture was a decisive variable.

### 2.2. Study Group

The study group consisted of multicultural parents (*n* = 100) with children aged 36–72 months living in the districts of Kyrenia, Nicosia, and Famagusta, where the population is concentrated in the Turkish Republic of Northern Cyprus (TRNC). The reason why parents with children in this age group were selected as participants was that this period is a time when children’s perceptions, values, and beliefs are rapidly shaped, cultural influences can be observed more clearly, and the effects of various cultural factors on creativity can be examined in detail (Zembat et al., 2019).

The study sample was created using the criterion sampling method. The inclusion criteria required that the parents in the nuclear families examined in the scope of the study should come from different cultures, have children aged 36–72 months, and live in one of the districts of Kyrenia, Nicosia, and Famagusta in the TRNC.

### 2.3. Ethical Procedures

Study approval was first obtained from the Eastern Mediterranean University Scientific Research and Publication Ethics Committee in accordance with the ethical procedures of the study during the data collection process. Data collection tools were assessed by three expert reviewers, all of whom are faculty members at three different state universities (one in the TRNC and two in Türkiye) with competence in creativity education. Before participating in the study, parents were informed about the subject and purpose of the research. They were told that the interview was voluntary, that the data obtained from the interview would be used solely for scientific purposes, that personal information would be kept confidential, and that they could withdraw from the interview at any time. After the participants signed the consent forms, interview appointments were organized with the participants.

### 2.4. Data Collection Process

A researcher-developed Demographic Information Form was used to collect descriptive information about the participating parents. It includes seven items containing the parents’ full names, age, educational status, and place of birth, place where they grew up, number of children in the family, birth order, and age of the child. The other data collection tool, a semi-structured interview form consisting of 10 open-ended questions, was developed by researchers to identify the cultural values families transmit while raising their children. The form was used in interviews with parents who were invited to participate through preliminary telephone interviews, with appointments scheduled for those who agreed to participate. Interviews were first conducted with parents who agreed to face-to-face interviews and subsequently with those who opted for online interviews. If children were present during the interview, their consent was also obtained; however, no individual interviews were conducted with children. Finally, home visits were conducted during which photographs were taken of the materials and resources parents used to support their children’s creativity. Only photographs were used as visual records. To ensure data diversity (triangulation), video and photo recordings were made of symbols—including informative memories, photographs, and cultural objects related to rituals, celebrations, dances, and games shared by parents with the researcher. Written and visual documents related to cultural elements—such as stories, memories, and fairy tales—that parents wanted to share were also recorded. Then, interviews with parents were conducted between 18 December 2023, and 25 March 2024. Each interview lasted 20–35 min on average. A total of 3043 min of interview data was obtained. Each researcher was assigned codes such as PA1, PB2 to protect the personal information of the parents participating in the study. The data obtained from the interviews were independently coded by both researchers. The Miles and Huberman (1994) inter-coder reliability calculation formula was applied to the parallel coding. The calculation revealed that the inter-coder agreement was 86%. The visual and written materials were analyzed using content analysis, with visual content analyzed specifically through visual content analysis.

## 3. Results

Demographic characteristics of the participating parents are shown in Table 1.

Most of the parents held a bachelor’s degree. Among them, 36 were aged 26–35 years, 51 were aged 36–45 years, 12 were aged 46–55 years, and one was aged ≥56 years. Most of them had one child. Regarding the children’s ages, 31 were three, 26 were four, five were five years old, and 53 were six years old (Table 1).

Regarding parents’ birthplaces, the most common country was TRNC (*n* = 22), followed by Turkey (*n* = 20), Iran (*n* = 6), Moldova (*n* = 6), Russia (*n* = 6), Ukraine (*n* = 5), Indonesia (*n* = 4), Pakistan (*n* = 4), the United Kingdom (*n* = 4), Kyrgyzstan (*n* = 4), Turkmenistan (*n* = 3), Belarus (*n* = 2), and others (Table 1).

Parents’ responses regarding their definitions of creativity were coded and combined to form 12 distinct themes. As seen in Table 2, most participants focused on innovation, invention, problem-solving, and productivity. Examples of parents’ definitions of creativity are as follows:

P2: *Creativity, innovation. It is doing something like inventing (Theme 1).*

P27: *Creativity is doing, producing, or trying to produce something that has not been done or thought of before (Theme 5).*

P65: *The ability to express ourselves and our ideas in new ways (Theme 3).*

The metaphors used by parents to describe creativity, along with their explanations for these metaphors, were coded and grouped into six distinct themes and their subthemes.

Analysis and data showed that natural elements and abstract concepts were prominent in parents’ perceptions of creativity (Table 3). Creativity was most frequently associated with natural phenomena such as rain, the ocean, and space, highlighting themes of eternity, the source of life, and diversity. In the metaphors related to the arts, disciplines such as sculpture, painting, and music were associated with creativity as they require effort and reflect imagination. Parents defined child-rearing both as a product of creativity and a process requiring creative effort within the family context. They also viewed everyday activities such as cooking as a reflection of creativity. These findings revealed that parents perceived creativity as a concept intertwined with both universal phenomena and everyday experiences.

Examples of statements regarding the reasons for the metaphors parents used when defining creativity were as follows:

P1: *It allows creativity (Theme 1).*

P26: *Because our horizons are broad. You can’t see ahead. Everyone’s creativity is different. Everyone has different perspectives. So, it has no limits. Everything that people and all kinds of living things see and create is an example of creativity. It is like that in outer space. No one can see where it starts and ends (Theme 2).*

P69: *Because they think about very different things, they research very different things. Things no one else thinks about (Theme 3).*

Parents’ responses regarding activities they engage in at home, within the family environment, and in daily life to support their children’s creativity were coded and combined to form nine distinct themes and subthemes.

As seen in Table 4, the activities that parents engaged in to support their children’s creativity most commonly included daily routines such as reading books, providing a multilingual environment, and cooking. Among artistic activities, painting and drawing stood out, while performance-based activities such as music and dance also played an important role. Traditional practices such as singing lullabies and storytelling were noteworthy in the context of the transmission of culture. The use of educational materials appeared as an important method for supporting children’s cognitive development. Games such as Legos and puzzles, in particular, emerged as activities that encouraged creative thinking. Parents viewed sports activities as tools for developing teamwork, strategic thinking skills, and physical development. Examples of statements from parents regarding the activities they do to support their children’s creativity are as follows:

P1: *When they are young, we do more activities. We read lots of books in the first year. I read books only in Serbian. My uncle produces children’s books, that’s why I bring a lot of books from Serbia. Our children love playing with playdough. We love cooking with the kids. We make crepes, pancakes, and pizza. They love drawing (Theme 1).*

P12: *When I come home from work, we play games together, we draw his/her pictures together, and we spend time together until bedtime. We mostly paint pictures together (Theme 4).*

P25: *We go out to the garden and collect leaves. Then we make pictures with those leaves. We make dresses for girls. We collect stones, bring them home, and paint them. We draw pictures of flowers. We take old cardboard and make whatever he/she wants. We paint and cut. We make pastries. He/she helps me because I usually make pastries. We also make cakes and cookies. She loves it. I give both him/her and myself a container, and he/she makes his/her own dough/cake. He/she does it with great enthusiasm; this is something that really supports children’s creativity. It really supports their imagination (Themes 3 and 5).*

The data presented in Table 5 indicate that, among the activities parents preferred to improve their children’s creativity in social and outdoor settings, nature-based activities stood out prominently. Visits to parks and walks in nature were the most frequently used approaches, while interaction with natural elements such as the sea and animals were also perceived to play an important role in creative development. Family and friend visits, as well as group meal arrangements, were among the preferred practices for developing children’s social skills through social interaction. In terms of cultural and educational activities, artistic events such as courses and theater were prominent, while rarer activities such as museum visits provided valuable contributions to increasing cultural awareness. In physical activities, parents preferred sports such as ball games and swimming, while disciplines such as ballet and gymnastics were found to support both physical and creative development. Parents viewed participation in music and painting courses in the field of art as an important step towards developing children’s esthetic sensitivity and creative expression skills. Examples of activities parents do to support their children’s creativity are as follows:

P12: *We go for walks, go to the park, play ball, and do sports. We collect flowers, soil, and stones. We go to the mountains, watch the scenery. We love walking in the mountains. Sometimes we go to museums (Theme 1).*

P22: *He/she goes to an international school appropriate for his age group. He/she has an active environment at home and outside; we visit the family, his/her friends, and interesting places. I take him/her to see musical instruments in cafés. Sometimes he/she likes to play. We try to find courses for children, such as pottery or outdoor activities, I mean, outdoor education, in addition to classroom education. We do many things (Theme 3).*

P36: *Going to the park, sliding, swinging, jumping… They love jumping at Barış Park. We go to the zoo. We go to villages and feed the lambs; they love animals. We spend more time in nature, outdoors (Theme 4).*

Table 6 shows the activities parents engaged in with their children to transmit cultural heritage. The most prominent activities emphasized by parents were traditional celebrations and rituals. In addition to holidays and special occasion celebrations, which play a central role in preserving cultural identity, parents used these activities to strengthen social bonds. Practices related to culinary culture, such as cooking and family meals, also emerged as important tools for intergenerational transmission. The use of parents’ native languages and storytelling in these languages plays a critical role in preserving the native language and maintaining folkloric elements. Parents also employed traditional handicrafts and games as an effective method for transmitting the intangible elements of cultural heritage to future generations. Performance-based activities such as music and dance contributed to the vivid continuation of cultural expressions. Within the scope of values education, social norms—such as respect for elders and solidarity—were particularly emphasized within the family. Examples of parents’ statements regarding celebrations, rituals, traditions, and ceremonies that reflect their own culture and that they engage in with their children are as follows:

P25: *Decorations made from coconut leaves are very beautiful. I teach it to my children. In fact, they really love it. I teach it even to my husband (it is definitely done in religious celebrations). I show them our culinary culture. They wear our clothes. Now, in Bali, the situation is like this—there are no apartment buildings as we have here. Houses there are more like a bungalow. Think that this is your kitchen. Next to it is another bungalow, which is your living room, and another is your bedroom. The rooms are all separate on a plot of land. Inside, it is like the Salamis Ruins. There are stone structures like that. You go inside and see things there. It is not a closed space, not like a mosque. It is open, open-air (Theme 1).*

P27: *I teach dances from my own culture. We play music from Black Sea and dance the horon (Theme 3).*

P37: *I teach my native language. My mom is with me. We look after them together, 24 h a day, because they are twin boys. I speak Russian, my mom speaks Russian, my husband speaks Turkish, and my mother-in-law speaks Turkish. I want them to learn the games I played when I was a child, such as tag, hide and seek, jump rope, and hot potato. We grew up playing these games (Theme 2).*

Regarding parents’ objectives for carrying out cultural heritage activities, these practices served multi-layered social and cultural functions. The primary motivation was the preservation and transmission of culture. A significant portion of participants stated that they practiced these activities primarily to preserve their cultural values and teach them to future generations. Strengthening social bonds, ensuring family unity, and developing social relationships were among other important goals that parents frequently emphasized. Elements such as teaching traditions, fostering historical awareness, and transmitting social values were particularly prominent in terms of educational objectives. It was also noted that some of the activities served functional purposes such as entertainment and escape from daily routines. The transmission of religious and faith-based values was another fundamental objective identified by parents (Table 7). Examples of parents’ statements regarding the purpose of celebrations, rituals, traditions, and ceremonies that reflect their culture and are practiced with their children are as follows:

P7: *Children need to understand when they grow up. They should know who they were born to, where they came from. These are done so that they know exactly which language and which faith to adopt and decide for themselves. Even in nature, a cat comes from a big cat, and a dog comes from another dog. It is done so they know their family, their past (Theme 1).*

P77: *To transmit traditions and bring together families. I think traditions and rituals like these bring families together. Culture did not form overnight; it was formed as a result of geographical and human relationships. It is passed down from generation to generation. It is important to preserve these values (Theme 2).*

The data in Table 8, which detail the values and messages that parents aim to pass on through cultural activities, indicate that these practices reflect a multidimensional process of education and value transmission. Fundamental social values such as respect for elders and the environment were among the most emphasized topics, reflecting the importance that parents placed on preserving traditional values. Under the theme of cultural heritage and identity, the importance of connecting with the past and developing a sense of cultural belonging were notable objectives. Parents’ emphasis on unity and togetherness highlighted the importance of strengthening family bonds and social solidarity. In terms of education and development, aims such as fostering historical awareness and supporting mental development were particularly prominent. Universal values such as respect for differences and tolerance were also among the important messages that parents wanted to pass on. Examples of parents’ statements regarding the values and messages they wish to transmit through activities representing their own culture and that they practiced together with their children were as follows:

P1: *Whatever you experience when you were a child stays with you when you grow up. I remember the cultural activities of my childhood as my happiest and most peaceful days. They teach unity and togetherness, and I think that also provides them with these (Theme 1).*

P23: *I think they contribute to the child’s understanding of identity, sense of belonging, and purpose. And a sense of patriotism and a mission, you know, social responsibility, and for the benefit of society and the nation. I work in an international office here, so I see a lot of this in international students. They are very, very proud of their countries, their heritage, their history, and of course, every country is unique. But it is great to see this because this is our identity, and this is what we develop within (Theme 2).*

Three main approaches emerged based on the data in Table 9 regarding parents’ attitudes toward traditional elements in the process of transmitting culture. The first group of parents (*n* = 59) tended to preserve and transmit traditional elements as they are. These participants adopted a determined attitude toward showing loyalty to the past and not changing cultural elements. The second group (*n* = 42) tended to modify traditional elements, often explaining this as a necessity arising from living in different cultural environments. Parents adopting the fourth approach (*n* = 16) tended not to transmit cultural elements for various reasons. Factors such as the passing of time, technological barriers, and living conditions were prominent in this group. A small group (*n* = 8) adopting the hybrid cultural transmission approach strived to create a new synthesis by blending elements from different cultures. Examples of parents’ attitudes toward traditional elements in activities representing their own culture, and they were engaged in with their children are as follows:

P19: *I pass on everything from my own culture as it is (Theme 1).*

P25: *Now, of course, we have to change some things from our time (Theme 2).*

Table 10 represents the roles of parents in organizing cultural activities. As seen, mothers were more actively involved in this process. In many families, this responsibility was assumed by mothers primarily as they spend more time at home, while fathers’ work schedules limited their participation. Although fathers took the lead less frequently, this responsibility was shared equally in some families. Notably, fathers assumed greater responsibility, especially when mothers who worked faced time constraints. The participation of grandparents or the immediate family circle generally depended on the parents’ workload.

As indicated in Table 11, the cultural and religious celebrations that parents share with their children reflect notable diversity and richness. Among religious holidays, Ramadan and Eid al-Adha stood out, with traditions such as wearing special clothing, visiting relatives, and sharing food playing an important role. Practices such as egg painting, going to church, and preparing special meals were commonly observed in Christian celebrations such as Easter and Christmas.

International events and festivals included various fruit and agricultural festivals, as well as culturally rooted special days. Special dates such as birthdays, wedding anniversaries, and Mother’s and Father’s Day were celebrated within the family through a variety of activities. Examples of statements related to celebrations and/or rituals specific to parents’ culture and parents perform with their children are as follows:

P3: *We also have Mother’s Day and Father’s Day, but they are different here. For example, on Mother’s Day, the children tie my feet with a string and ask me for a gift; I give them a gift. I was very surprised that gifts are given to parents here. I buy gifts for my spouse’s mother, but in my country, mothers buy gifts for their children. In our culture, if New Year’s Day is Sunday, Mother’s Day is celebrated on Saturday, one day before. Father’s Day is celebrated on the following Saturday (Theme 4).*

P9: *Russian culture has a pancake festival. It is called the Maslenitsa Festival, and it is very beautiful. It is held at the end of winter and the beginning of summer. It is held to say goodbye to winter. A large puppet is made of straw. It is made in the square. Stalls are set up, tea is made, and pancakes are made. The puppet should absolutely be in the middle. It is burned at a certain time; it must be burned. When this puppet burns, it means winter is over, and spring comes (Theme 2).*

Data in Table 12 indicate varying levels of children’s participation and motivation in cultural celebrations and rituals. Active participation was the most common approach, with children displaying behaviors such as helping, practicing together, and imitating what they observed during this process. The desire for fun and learning stood out as important motivational factors in children’s participation in these activities.

Children who participated passively predominantly exhibited behaviors such as observing, listening, and showing curiosity. These children were generally involved in the process for learning purposes or at the direction of their parents. However, those who did not participate in these activities did so mainly due to their own preferences or their parents’ lack of guidance. Examples of parents’ statements regarding the roles their children undertake in celebrations and rituals and the reasons behind them are as follows:

P18: *They are just having fun. They are trying to take advantage of the opportunities we offer them. That is, if we can offer (Theme 1).*

P44: *They wear clothes. They participate in the decorations. Sometimes they participate in cooking (Theme 2).*

P99: *I observe him/her helping their mother (Theme 3).*

Based on the data in Table 13, parents’ roles in cultural celebrations and rituals primarily emphasized being cultural transmitters and preparers of the environment. Most of the parents actively took on the responsibility of teaching and explaining cultural heritage to their children during these events. The underlying motivation for this role was the desire to ensure cultural continuity and pass on traditional values to future generations.

The role of preparing the setting reflected parents’ responsibility for creating the physical and emotional atmosphere of celebrations. This role involved ensuring the necessary conditions for cultural events were properly organized. Roles that parents assumed less frequently, such as providing support and serving as role models, aimed to provide guidance and behavioral models that children needed during this process.

The most prominent reason parents assumed these roles was the desire to ensure cultural transmission and teaching. These roles, regarded as a natural responsibility of parenthood, were also shaped by social expectations and the requirements of the traditional family structure. Examples of statements regarding the roles parents undertook in celebrations and rituals, and their reasons are as follows:

P1: *I serve as a role model (Theme 4).*

P33: *I make preparations. My child and I write letters by hand to our family elders. We send them. I say, “Grandma and Grandpa will be very happy when they see them (Theme 1).*

## 4. Discussion

The results of the study showed that multicultural parents did not view creativity as limited only to artistic skills, but also associated it with broader domains such as nature, the universe, abstract concepts, and family bonds. This indicated that creativity was intertwined with various everyday experiences and life events. In addition, parents employed different contexts when supporting their children’s creativity. Parents’ definition of creativity using a variety of metaphors across a wide range of areas showed that they adopted a broad and flexible approach to their children’s creative development. These results provide important clues about how cultural contexts shape creativity and show that parents develop multifaceted strategies when they support their children’s creativity. A review of the literature shows that the definition of creativity differs according to both time and cultural context. According to Walia (2019), the definition of creativity has evolved over time through various perspectives and approaches. Initially, it was seen as a process emerging through motivation or blind variation, i.e., random trial and error. However, in subsequent years, it has been considered a complex process involving different ways of thinking, the ability to discover new problems, and the journey from an opinion to a tangible outcome. Csikszentmihalyi (2014), explains that creativity is a multidimensional concept emerging as a result of interactions between individual characteristics and social contexts and notes that the definition and perception of creativity can vary significantly depending on cultural contexts.

Parents supported their children’s creativity through various activities, both at home and in social environments. Supporting children’s creativity develops their problem-solving skills and allows them to become individuals who can express themselves freely. Therefore, the home environment should be equipped with diverse stimuli encouraging the child’s imagination and enabling them to explore and experiment (Yazgın & Yazgın, 2023). Pugsley and Acar (2020) reported that parents’ attitudes and values towards creativity and a creative home environment were significantly and positively related to their support of their children’s creative characteristics.

Parents highlighted activities such as learning and preservation of the mother tongue, storytelling and the transmission of folkloric stories, and the strengthening of cultural identity through language and stories under the theme of “language and storytelling”. Multicultural families use transmission of language to develop their children’s creativity, strengthen their cultural identity, and allow them to communicate with members of the extended family. In addition, they adopted this method to pass on their culture to their children and to preserve and protect their cultural language. The study found that multicultural parents employed a common method for transmitting language. From the moment their children were born, the mother and father communicated with them only in their native languages, while the spouses specified a common language between themselves, in which they communicated. The children were exposed to three separate languages within the family: the mother’s native tongue, the father’s native tongue, and the common language used in family conversations. In her study on Multicultural Family Language Policies, Danjo (2021) noted she had observed the same situation. The One Parent One Language policy, one of the most common family language strategies in multicultural families, involves each parent consistently using their own native language when talking with their children. Multicultural families aim to empower their children with linguistic competence and strengthen their cultural identities by adopting such language strategies. However, children often go beyond these boundaries and creatively use translanguaging. Santello (2022) approaches translanguaging as a holistic construct, composed of different linguistic resources formed within social interactions. Children communicate by blending two, three, or four languages within a single sentence, and this plays an important role in their process of making sense of their multilingual world. This process is an approach supporting creativity and linguistic skills through the flexible and dynamic use of linguistic resources.

Parents’ responses showed that the celebrations and rituals they performed with their children played an important role in both preserving cultural identity and supporting their children’s creativity. The results revealed that children of multicultural parents participated in different cultural celebrations and rituals. Çelik et al. (2016) investigated the association between multiculturalism and creativity by examining the effect of exposure to various cultural values on divergent (different) thinking skills. Their results revealed that when individuals were exposed to fundamental cultural values differing from their own, their cognitive flexibility increased, and this developed divergent thinking—a critical element in creativity. In other words, the more deeply and openly individuals participate in multicultural experiences, the more their creativity is strengthened. Birinci and Grin (2023) examined the effects of multilingualism and multicultural experiences on creativity and found that multilingualism and multicultural exposure, in particular, increased creative thinking skills. Another study reported that individuals’ diverse cultural backgrounds not only increased individual creativity but also made significant contributions to collective creativity within a team (Tadmor et al., 2012).

Parents emphasized fostering unity and togetherness, families spending time together, and people socializing under the theme of “Strengthening Togetherness and Social Bonds.” This indicates that cultural activities and traditions are essential tools for strengthening bonds not only individually but also socially. It was found that parents attached great importance to preserving cultural values, transmitting these values to future generations, and ensuring that cultural roots are not forgotten under the theme of “Cultural Transmission and Preservation.” These findings demonstrate that parents play an active role in the sustainability of culture and perform a critical function in transmitting cultural heritage to future generations.

According to the research results that parents’ goals for participating in cultural activities and the messages they want to transmit to their children mostly overlapped and were organized around five main themes. Additionally, cultural activities were seen as important tools not only at the individual level but also for strengthening social bonds and preserving cultural identity. Parents’ role in these processes showed that they serve a critical function in transmitting cultural and religious values, as well as social bonds and tolerance, to future generations. In this context, the results of this study are consistent with those of Rogoff (2003), who reported that prosocial behaviors are transmitted at an early age through parents within a cultural framework, children’s socialization processes are shaped within the framework of cultural practices and community norms, and prosocial behaviors (such as helping, sharing, and supporting others) are transmitted within this context. Cultures strengthen different skills and cognitive processes. Cultures also have an impact on values. The results of Storme et al.’s (2015) research also showed that culture has an impact on the structure of creative ability. According to Coppens and Rogoff (2022), parents teach their children not only cultural identity and values but also prosocial behaviors that foster responsibility toward society and others through cultural activities and rituals. They reported that prosocial behaviors were taught to children from an early age within a cultural context and that these behaviors were shaped based on cultural differences. The researchers conducted their study with Mexican-origin and European-origin mothers and children. They observed that Mexican-origin children were more likely to volunteer to help with household chores, whereas European-origin children rarely did so and usually helped only under adult supervision and within structured arrangements. Based on their observations, the researchers noted that cultural differences were the reason for the variation in children’s behaviors.

Most parents who participated in the study tended to preserve and transmit cultural heritage without changing it. Modifying traditional elements: Some parents found it appropriate to modify certain traditional elements when transmitting their cultural heritage to their children. These modifications stemmed from factors such as parents living in different cultural environments and distance from their traditions. Families living in different cultural environments adapt these elements to their own experiences and surroundings. This result is consistent with Leontiev’s (2009) activity theory, suggesting that individuals’ activities are shaped within social and cultural contexts. People engage in activities in line with the norms, values, and expectations of their culture. Regardless of the conditions under which these activities occur or the forms they take, they cannot be considered separately from social relations and the life of society. Hybrid cultural transmission: Parents making bicultural transmission and adaptation passed on elements of multiple cultures to their children by combining them. This approach resulted from parents’ experiences of living in different cultural environments. Hybrid cultural transmission contributes to children developing a multicultural identity. A study exploring how parents in multicultural families shared their cultural elements with their children found that parents invested in actively learning about and supporting both cultures by working together to help their children adopt and develop their cultural identities (Seider et al., 2023). Not transmitting any traditional elements: Some parents did not pass on cultural elements to their children or do not care about these elements. This attitude stemmed from factors such as the changing times, the desire to raise global citizens, reluctance to transmit cultural elements, technological barriers, and the inability to create a traditional environment. Additionally, some parents experienced a lack of motivation to transmit to their children the traditions they themselves had experienced. Crippen and Brew (2013), who examined the cultural adaptation strategies of cross-cultural parents, drew similar conclusions. Leung and Chiu (2010) aimed to examine the role of multicultural experiences in creative performance, specifically investigating the short- and long-term effects of exposure to a foreign culture on creative performance. In three of their studies, they found that multicultural experiences positively affected creative cognitive processes. Researchers emphasize that while it is known that culturally sensitive social-emotional learning has positive effects on improving students’ academic and emotional resilience, there is still insufficient scientific evidence on how it is implemented in rural school settings. Lin et al. (2025), in their research conducted with early childhood teachers in rural schools, highlighted the positive contributions of supporting culturally sensitive social-emotional learning practices at home, within the family, to school practices.

Results showed that gender roles and parents’ heavy workloads impacted the distribution of organizing children’s activities. Peng (2022) examined how mothers and fathers take on different roles in childcare within the digital environment and found that digital technologies intensified childcare responsibilities, with mothers, in particular, taking on a larger share. The study showed that gender roles played a role not only in physical tasks but also in parenting tasks carried out via digital platforms. Craig and Mullan (2011) conducted a study exploring how parents shared childcare roles and examined cross-country differences in sharing. They found that mothers generally spent more time dedicated to childcare, but fathers were also increasingly involved. In some countries, the time that fathers spent on childcare was more, while mothers’ time remained constant. How childcare was shared varied depending on cultural norms, the existence of social policies, and economic conditions.

The present study examined parents’ responses regarding the roles that their children assumed in celebrations and rituals under three main themes: passive participation, active participation, and no participation. Under the theme of passive participation, parents generally stated that their children displayed behaviors such as observing and listening to what was being said. The motivations behind this were curiosity, a desire to learn, and parents’ expectations. Most of the parents said their children actively participated in cultural activities. The active participation theme emphasized parents’ engagement with their children, helping them, and focusing on fun. While parents supported their children’s active roles in these activities, they also stated that the children were motivated by learning and enjoyment. Additionally, parents’ desire to be with their children and their sense of responsibility were also important factors driving this participation. Very few parents reported that their children did not participate in cultural activities. The theme of no participation indicated that, in some cases, parents did not want their children to participate in celebrations and rituals or preferred to keep them away from such activities. This was due to parents’ preferences, which shaped their decisions. These results showed the complexity of the dynamics affecting children’s participation in cultural activities and various motivations. Parents’ influence on their children’s participation in celebrations and rituals plays an important role in the transmission of cultural values and children’s social and emotional development. Rogoff (2016) proposes the “Learning by Observing and Pitching In” (LOPI) approach. LOPI emphasizes that children learn best through observation, social interaction, and participation in the activities of their community. The results of the present study can be explained by the concept of “Guided Participation” developed by Rogoff (2016), which refers to parents’ participation focused on doing, helping, and having fun with their children, and is a model that encourages children to learn from more experienced individuals and take an active role in the process.

This study examined parents’ responses to the roles they undertook in their children’s celebrations and rituals under five main themes: cultural transmitter, preparing the setting, providing support, being a role model, and taking to visits. The cultural transmitter theme highlighted that most of the parents assumed the role of teaching and explaining cultural values to their children. Parents reported that their primary goals were to ensure cultural transmission and provide teaching. Additionally, the sense of responsibility inherent in parenthood and the desire to guide their children also influenced this process. This result is consistent with that of Leguina et al. (2022), who examined the role of parents in children’s participation in cultural activities and how different forms of parents’ cultural capital were reflected in children’s cultural behaviors.

The findings of this study showed that parents played an active role in children’s participation in celebrations and rituals and assumed significant responsibility for the transmission of cultural values. In particular, parents’ access to cultural activities and social networks contributed to the enlargement of children’s cultural experiences.

## Figures and Tables

**Table 1:** Distribution of Participants by Demographic Characteristics.

Personal Features	Subcategories	*n*
**Educational status**	High school	19
	Associate degree	5
	Bachelor’s degree	49
	Master’s degree	14
	Doctorate	13
	**Total**	**100**
**Age**	26–35	36
	36–45	51
	46–55	12
	≥56	1
	**Total**	**100**
**Number of children**	1	54
	2	39
	3 and more	7
	**Total**	**100**
**The birth order of the child participating in the study**	1	66
	2	43
	3	4
	4	2
	**Total**	**115**
**Age of the children participating in the study**	3	31
	4	26
	5	05
	6	53
	**Total**	**115**
**Countries where the parents were born**	TRNC	22
	Türkiye	20
	Iran	6
	Moldova	6
	Russia	6
	Ukraine	5
	Indonesia	4
	Pakistan	4
	United Kingdom/London	4
	Kyrgyzstan	4
	Turkmenistan	3
	Belarus	2
	Tataristan	2
	Southern Cyprus	2
	Greece	1
	Germany	1
	Bangladesh	1
	Bulgaria	1
	Africa/Cameroon	1
	Bosnia and Herzegovina	1
	Slovakia	1
	Romania	1
	Azerbaijan	1
	Serbia	1
**Countries where the parents grew up**	TRNC	25
	Türkiye	20
	Moldova	6
	Iran	6
	Ukraine	5
	Russia	5
	Kyrgyzstan	4
	Pakistan	4
	Indonesia	4
	Turkmenistan	3
	United Kingdom	3
	Southern Cyprus	2
	Belarus	2
	Tataristan	2
	Bulgaria	1
	Serbia	1
	Azerbaijan	1
	Germany	1
	Bangladesh	1
	Africa/Cameroon	1
	Bosnia and Herzegovina	1
	Slovakia	1
	Romania	1
**Place of participation**	Kyrenia	68
	Famagusta	14
	Nicosia	18

**Table 2:** Parents’ Definitions of Creativity.

Category	Creativity Tanımları
Themes *	*n*
Innovation and Invention	54
Problem Solving and Productivity	15
The Ability to Think Differently by Thinking Outside the Box	9
Imagination and Inspiration	7
Originality	5
Art and Esthetics	5
Education and Development	4
Self-Expression	4
God’s Creation	4
Bringing Ideas to Life	3
Innate Talent	3
Courage and Open-Mindedness	2

* Participants selected more than one theme.

**Table 3:** Metaphors Parents Use to Describe Creativity and the Reasons behind Their Usage.

Category			Metaphors for Definitions of Creativity	
Themes	Subthemes *	*n*	Reasons	*n*
**Metaphors related to art and design**	Painting/Drawing	2	Refreshes the mind	2
	Theater play/Drama	2	Enables the use of imagination	2
	Sculpture	2	Requires effort and detail	2
	Colors	2	Bringing happiness/Revealing beauty	2
**Metaphors related to nature and the universe**	Rain/Water	7	Creation/source of life (*n* = 5)/usefulness (*n* = 2)	7
	Ocean/Sea	4	Eternity	4
	Space	4	Eternity	4
	Rainbow	3	Colorfulness	3
	Earth	3	Perfection (*n* = 2)/Eternity (*n* = 1)	3
	Seed	2	Being the beginning of everything	2
	Path	2	Including surprises/boundlessness	2
	Sun	2	Being useful	2
	Life	4	Being a product of creativity (*n* = 2)/Uniqueness (*n* = 2)	4
**Abstract and symbolic metaphors**	Beauty	2	What is created is beautiful	2
	Dream/Fairy tale	2	Extraordinariness	2
	Happiness	2	Creativity brings happiness	2
**Metaphors related to family and child**	Child/baby	5	Raising requires creativity/effort (*n* = 3)/doing creative things (*n* = 2)	5
	Ocean/Sea	5	Being reassuring (*n* = 2)/requiring creativity (*n* = 3)	5
	Space	4	Coming from nothing (*n* = 2)/requires effort (*n* = 2)	4
**Metaphors related to games and intelligence**	Playing games	2	Product of creation (*n* = 2)	2
	Puzzles	2	Solving it requires creativity.	2
**Other**	Cooking	6	Diversity (*n* = 3)/desire for creativity (*n* = 3)	6
	Everything you see around you	6	Being a product of creativity	6
	Daydreaming	3	Opens doors to new things (*n* = 2)	3

* Participants selected more than one theme.

**Table 4:** Activities Parents Do at Home, in the Family Environment, and in Everyday Life to Support Their Children’s Creativity.

Category	Activities to Support Creativity	
Themes	Subthemes *	*n*
**Reading books**		40
**Multilingual environment and language teaching**		31
**Cooking and kitchen experiences**		25
**Artistic events**	Painting/drawing	42
	Music and dance/rhythm	16
	Crafts	5
**Creative games and games**	Legos	11
	Hide and seek/tag	3
**Cultural activities and traditions**	Singing lullabies	6
	Telling stories	4
	Teaching traditional handicrafts	4
**Educational materials and academic development**	Watching educational films	11
	Playing with educational toys	8
	Sending to a course/preschool	8
	Puzzles	6
	Teaching letters and numbers	5
	Play dough	2
**Sports activities**	Soccer	4
	Tennis	3
	Basketball	3

* Participants selected more than one theme.

**Table 5:** Activities Parents Engage in to Support Their Children’s Creativity Outside Home, in Extended Family or Social Settings.

Category	External Activities to Support Creativity	
Themes	Subthemes *	*n*
**Activities performed in nature and outdoors**	Taking them to parks/playgrounds	69
	Walk in nature	35
	Going to the beach	21
	Meeting animals	19
	Keeping them busy in the garden	15
	Having a picnic	14
	Taking outdoors	5
	Camping	4
**Social interaction and spending time with close friends and family**	Visiting friends/family	18
	Eating out	14
	Birthday parties	5
**Education and cultural events**	Sending to various courses	14
	Participation in theater or artistic events	5
	Visits to museums	2
**Physical activities and sports**	Playing ball	13
	Swimming	8
	Going to ballet/gymnastics	7
	Ice skating	5

* Participants selected more than one theme.

**Table 6:** Activities That Represent Parents’ Culture and Parents Engage in Together with Their Children.

Category	Children’s Experiencing and Learning About Cultural Heritage	
Themes	Subthemes *	*n*
**Traditional foods and culinary events**	The tradition of cooking and spending time in the kitchen	11
	Passing on memories and values related to family meals	5
	Learning and making recipes from different cultures	4
**The tradition of language and storytelling**	Learning and preserving the mother tongue	16
	Storytelling and passing on folkloric tales	7
	Strengthening cultural identity through language and stories	4
**Traditional celebrations and rituals**	Celebrating holidays, festivals, and special days	29
	Explaining the importance of celebrations and strengthening social bonds	15
	Keeping religious and cultural rituals alive within the family	10
**Handicrafts and games**	Playing traditional games and organizing fun activities	4
**Music and dancing**	Learning cultural music and dances	5
**Values education**	Children not speaking in the presence of elders/Kissing elders’ hands	10
	Mutual aid/love	5
	Togetherness/solidarity	4

* Participants selected more than one sub-theme.

**Table 7:** The Purpose of Activities Representing Parents’ Culture that They Engage in with Their Children.

Category	The Purpose of Children Experiencing and Learning About Cultural Heritage	
Themes	Subthemes *	*n*
**Strengthening unity and social bonds**	Enabling unity and togetherness	12
	Families’ spending time together	7
	Socialization	7
**Cultural transmission and preservation**	Preserving and teaching cultural values	35
	Transmitting culture to future generations	13
	Not forgetting cultural roots	5
**Education and learning**	Teaching traditions and customs	7
	Teaching culture	5
	Increasing historical and cultural awareness	4
	Teaching social values	4
**Entertainment**	Having fun and enjoying special occasions	8
**Transmission of values regarding religion and religious belief**	Living and sharing beliefs	5
	Transmitting beliefs and rituals	5

* Participants selected more than one sub-theme.

**Table 8:** Values and Messages Parents Want to Transmit Through Cultural Activities with Their Children.

Category	The Message Wanted to Be Given	
Themes	Subthemes *	*n*
**Unity and togetherness**	The importance of spending time with family and community	24
	Strengthening family ties	7
	The importance of sharing/supporting	5
**Cultural heritage and identity**	Transmission of cultural values	32
	Respect for the past	7
	Developing a sense of identity and belonging	4
**Values and ethics**	Teaching respect for elders, family, and the environment	35
	Human Values: Emphasizing being a good person, honesty, and morality	21
**Education and development**	Historical Awareness: Children learning their history and understanding the importance of the past	26
	Acceptance of and respect for different cultures and beliefs	9
	Supporting children’s learning processes and encouraging their mental development	5
**Tolerance/respect for differences**	Respect for Diversity: The importance of different cultures and beliefs coexisting	5

* Participants selected more than one sub-theme.

**Table 9:** Parents’ Attitudes towards Traditional Elements in Cultural Activities Shared With Their Children.

Category	Attitude Towards Traditional Elements	
Themes	Subthemes *	*n*
**Preserving traditional elements**	Transmitting as is, without changing anything	24
	Desire to preserve tradition	13
	Fidelity to the past	12
	Not finding a change appropriate	10
**Modifying traditional elements**	Changing some things	42
	Distance	13
	Living in a different culture	10
**Hybrid cultural transmission**	Bicultural transmissions and changes	4
	Living in different cultural environments	4
**Not transmitting any traditional elements**	Not transmitting or caring for cultural elements	16
	Change in time/raising global citizens	11
	Unwillingness	7
	Technological barriers	5
	Inability to create a traditional environment	3
	Not having time	3
	Lack of desire to share experiences	3

* Participants selected more than one sub-theme.

**Table 10:** Parents’ Opinions on Which Parent Undertakes More Responsibility for Organizing Activities Parents’.

Category	Attitudes Toward Activities	
	Themes *	*n*
**Father**	**Mother undertakes**	**27**
	***Reason***	
	Being busy/Having no time	24
	Mom being home more	14
	**Together**	**11**
	***Reason***	
	Sharing responsibilities	11
	Undertake themselves	**11**
**Mother**	Undertake themselves	**34**
	***Reason***	
	Spouse’s employment	19
	Spending more time at home	9
	Spouse’s lack of interest	5
	Better understanding of one’s own culture	5
	**Together**	**11**
	***Reason***	
	Sharing responsibilities	11
	**People other than parents (immediate circle)**	**5**
	***Reason***	
	Being busy/Having no time	5

* Participants selected more than one sub-theme.

**Table 11:** Cultural Celebrations and/or Rituals that Parents Share with Their Children.

Category	Cultural and Religious Holidays/Rituals	
	Subthemes *	*n*
**Religious holidays and rituals**	**Ramadan and Eid al-Adha**	**23**
	Wearing special clothing	23
	Fasting	10
	Visiting relatives/friends	10
	Collecting candy/chocolate	4
	Going to mosques	8
	Praying	4
	Helping the poor	4
	Cooking special meals	3
	**Easter and Christmas**	**22**
	Painting eggs	22
	Going to church	15
	Praying	14
	Cooking special meals	14
	Wearing special clothes	10
	Fasting	4
	Telling old stories	3
	**December 19 Saint Nicholas**	**3**
**National holidays and events**	**April 23 National Sovereignty and Children’s Day**	**10**
	**May 19 Commemoration of Atatürk, Youth and Sports Day**	**5**
	Aircraft/sound and light shows	10
	**Independence Days**	**5**
	Wearing local clothing	5
**Cultural and seasonal celebrations**	**Nowruz feast**	**10**
	Making special meals	5
	Wearing costumes	5
	Preparing 7 foods that start with the letter “s”	5
	Making sweets (samanu)	3
	**Halloween**	**3**
	**New Year’s celebrations**	**10**
	Decorating a tree	8
	**Sabantuy Festival**	**2**
	Gift giving	2
	Wearing local clothing	2
	Organizing entertainment activities	2
	**Olive, Potato, Tulip, Orange Festivals**	**5**
	**International events and festivals**	**5**
	**Maslenitsa**	**3**
	**Yalda Night**	**4**
	Preparing various red fruits	4
	**Wednesday Suri**	**4**
	Organizing shows and entertainment	4
**Special occasions and family celebrations**	**Valentine’s Day and Women’s Day**	**5**
	**Birthdays**	**4**
	**Mother’s Day and Father’s Day**	**3**
	**Wedding ceremony/anniversary**	**2**
	Gift	5

* Participants selected more than one sub-theme.

**Table 12:** Reasons Behind the Roles Parents Assume in Their Children’s Celebrations and Rituals.

Category	The Roles Children Assume	
Themes	Subthemes *	*n*
**Passive participation**	Observation	6
	Listening to what is being said	3
	***Reason***	
	Curiosity	5
	To learn	4
	Mom/dad wanted me to	4
**Active participation**	Help	30
	Do it together	16
	Imitate what you see	15
	Do what you are told	15
	Participate for fun	10
	***Reason***	
	They are children	21
	To learn	17
	To be happy/have fun	16
	Because their mother/father wants them to	16
	To be with their mother/father	10
	Because they feel responsible	4
**No participation**	Do/does not participate	**5**
	***Reason***	
	Parents’ request	3

* Participants selected more than one sub-theme.

**Table 13:** Roles That Parents Assume in Celebrations and Rituals and the Reasons behind Them.

Category	The Roles That Parents Assumed
Themes *	*n*
**Cultural transmitter (Teaching and narrating)**	**43**
**Preparing the setting**	**39**
**Providing support**	**8**
**Being a role model**	**8**
**Taking for visits**	**7**
***Reason***	
Ensure/teach cultural transmission	31
They are a parent	18
It is seen this way	8
Their role as a guide	7
They feel responsible	5

* Participants selected more than one sub-theme.

## Data Availability

Data is unavailable due to privacy or ethical restrictions.
